# Ratings of the Effectiveness of Nutraceuticals for Autism Spectrum Disorders: Results of a National Survey

**DOI:** 10.3390/jpm11090878

**Published:** 2021-08-31

**Authors:** James B. Adams, Anisha Bhargava, Devon M. Coleman, Richard E. Frye, Daniel A. Rossignol

**Affiliations:** 1 School of Engineering of Matter, Transport, and Energy, Arizona State University, P.O. Box 876106, Tempe, AZ 85287, USA; devon_marie@live.com; 2 Columbia Mailman School of Public Health, 722 W. 168th St., New York, NY 10032, USA; abharg25@asu.edu; 3 Section of Neurodevelopmental Disorders, Division of Neurology, Barrow Neurological Institute at Phoenix Children’s Hospital, 1919 E Thomas Rd., Phoenix, AZ 85016, USA; rfrye@phoenixchildrens.com; 4 Rossignol Medical Center, 24541 Pacific Park Drive, Suite 210, Aliso Viejo, CA 92656, USA; rossignolmd@gmail.com

**Keywords:** autism, autism spectrum disorder, nutraceuticals, survey, vitamins, minerals, B12, folinic acid

## Abstract

Autism spectrum disorder (ASD) often involves a wide range of co-occurring medical conditions (“comorbidities”) and biochemical abnormalities such as oxidative stress and mitochondrial dysfunction. Nutritional supplements (“Nutraceuticals”) are often used to treat both core ASD symptoms and comorbidities, but some have not yet been formally evaluated in ASD. The potential biological mechanisms of nutraceuticals include correction of micronutrient deficiencies due to a poor diet and support for metabolic processes such as redox regulation, mitochondrial dysfunction and melatonin production. This paper reports on the results of the National Survey on Treatment Effectiveness for Autism, focusing on nutraceuticals. The Survey involved 1286 participants from across the United States. Participants rated the overall perceived benefits and adverse effects of each nutraceutical, and also indicated the specific symptoms changed and adverse effects. From these ratings the top-rated nutraceuticals for each of 24 symptoms are listed. Compared to psychiatric and seizure medications rated through the same Survey, on average nutraceuticals had significantly higher ratings of Overall Benefit (1.59 vs. 1.39, *p* = 0.01) and significantly lower ratings of Overall Adverse Effects (0.1 vs. 0.9, *p* < 0.001). Folinic acid and vitamin B12 were two of the top-rated treatments. This study suggests that nutraceuticals may have clinical benefits and favorable adverse effect profiles.

## 1. Introduction

Autism spectrum disorder (ASD) is a complex neurodevelopmental disorder involving core problems in social communication and repetitive behaviors and affects about 2% of children in the United States [1]. A number of medical conditions cooccur with ASD (termed “comorbidities”), including intellectual disability [2], epilepsy [3], gastrointestinal disorders (such as constipation and diarrhea) [4], sleep disorders [5], attention deficit disorder [5], anxiety [5], and irritability, self-injurious behavior, and depression [6]. Other studies have reported biochemical abnormalities, including problems with methylation pathway insufficiency [7,8], insufficient production of melatonin for sleep [9], mitochondrial dysfunction [10,11] and oxidative stress [12,13]. Currently, there are no FDA-approved medications for treating the core symptoms of ASD (social communication and restricted/repetitive behaviors), although there are two FDA-approved medications for treating the associated symptom of irritability [14].

Compared to typically developing (TD) children, feeding difficulties are common in children with ASD and include food refusal, eating a limited variety of foods and having more problems with mealtime behavior [15], and they may have nutrient-poor diets [16]. One meta-analysis of 17 prospectively controlled studies reported significantly more feeding problems in children with ASD compared to controls (odd ratios 5.11, 95% CI 3.74–6.97) and significantly lower intake of calcium and protein in the ASD group [17]. A systematic review of 29 studies reported that feeding problems were associated with impaired sensory processing, perception, more rigidity and challenging behaviors [18]. A prospective, randomized controlled trial of a comprehensive dietary and nutritional intervention found that a combination of six treatments (vitamins/minerals, essential fatty acids, Epsom salts, carnitine, digestive enzymes, and a healthy low-allergen diet) led to significant improvements over one year in autism symptoms, developmental age, and non-verbal IQ compared to controls [19].

Because of potential deficiencies of nutrients (often related to feeding problems) and biochemical abnormalities (e.g., oxidative stress, mitochondrial dysfunction, methylation problems, among others) reported in individuals with ASD, a number of studies have investigated the use of vitamins, minerals, and other nutritional supplements (hereafter termed “Nutraceuticals”). The use of nutraceuticals is typically considered a form of complementary and alternative medicine (CAM), although many nutraceuticals are based on the science of nutritional biochemistry and target deficiencies and biochemical problems. Owen-Smith et al. (2015) conducted a survey of 42 CAM treatments used in ASD and reported 88% of participants had been treated with at least one CAM treatment [20]. Frye et al. (2011) surveyed the effectiveness of seizure treatments (including nutraceuticals) in 733 children with ASD and seizures compared to 290 controls and reported some CAM treatments (such as vitamin B6, magnesium, taurine, and vitamin B12) were rated as helpful for treating seizures [21]. In addition, a large online survey was conducted by the Autism Research Institute (the “Parent Ratings of Behavioral Effects of Biomedical Interventions Survey”) [22]. This survey of 27,000 parents of individuals with ASD rated the effectiveness of 84 various medications, supplements, and diets, using a six-point scale from ‘‘made worse’’ to ‘‘made better”; a number of treatments were reported as beneficial, including methylcobalamin (MB12), melatonin, digestive enzymes, fatty acids, cod liver oil, vitamin B6, zinc, magnesium, folic acid, vitamin C, and vitamin A [23]. Although these surveys focused on the overall effectiveness ratings for medications and nutraceuticals used in ASD individuals, most of these studies did not utilize a separate rating scale for the benefits and adverse effects (AEs) and did not obtain information on the effects of these treatments on specific symptoms of ASD.

Some of the medical comorbidities and biochemical abnormalities reported in individuals with ASD might improve with nutraceuticals. For example, randomized clinical trials for ASD have demonstrated the efficacy of melatonin supplementation [9], folinic acid [24,25,26], vitamin/mineral supplements [27,28], comprehensive nutritional interventions [19], N-acetyl cysteine (NAC) [29], and sulforaphane [30].

This paper presents the results of a national survey (the “National Survey on Treatment Effectiveness for Autism”) in individuals with ASD and contains more extensive assessments of the treatment effects on specific behaviors and AEs of nutraceuticals in ASD. A previous paper from this Survey reported on the results for psychiatric and seizure medications [31].

## 2. Materials and Methods

The research team created the “National Survey on Treatment Effectiveness for Autism” (from now on referred to as “the Survey”) and obtained reviews by families of children/adults with ASD and experts in a variety of fields who treat individuals with ASD. This study was approved by the Institutional Review Board of Arizona State University (STUDY00003766). The Survey was advertised to families of individuals with ASD across the country with the assistance of over 50 autism organizations (see Acknowledgements). A full explanation of the Survey creation and distribution can be found in the previous paper [31]. The Survey obtained general medical history and the use of psychiatric and seizure medications, general medications, nutraceuticals, diets, therapies, and information on Kindergarten through grade 12 education. This paper reports data only on the nutraceutical section from Survey responses from 1710 people (of which 1286 (75.2%) rated the effects of nutraceuticals); additional responses were collected since the analysis reported in the previous paper. The exact diagnosis of the individual with ASD was queried using the following categories: autism, Asperger’s syndrome, autism spectrum disorder, high-functioning autism, pervasive developmental disorder not otherwise specified (PDD-NOS), no current diagnosis but was previously on the autism spectrum, and “other” in order to capture both DSM-IV and DSM-5 diagnostic categories. These diagnoses were reported by the participant, but not verified in this study since it was an anonymous survey.

The Survey was divided into sub-sections for various types of nutraceuticals (amino acids, vitamins, etc.). At the beginning of each sub-section, the Survey asked what nutraceuticals the participant had taken (from a list of 123 nutraceuticals found in Table S1). For each nutraceutical taken, the Survey asked the participant to rate the overall perceived benefit of the nutraceutical (no benefit = 0, slight benefit = 1, moderate benefit = 2, good benefit = 3, great benefit = 4), the primary symptoms benefited (if any), the overall AE of the nutraceutical (no adverse effect = 0, mild adverse effect = 1, moderate adverse effect = 2, severe adverse effect = 3), and the specific symptoms that were adversely affected (if any). Table 1 shows the symptom list from which participants could select (they could select one or more for each treatment). Finally, the Survey asked for the overall average effect of all nutraceuticals (on a 7-point scale ranging from “much better” to “much worse”). Only treatments with 20 or more responses were included in this analysis. It should be noted that the ratings are the perceived benefit of the evaluator (primarily a caregiver or sometimes the person with ASD), and not ratings by a clinician or physician, which is a limitation of the study.

For each treatment, the top 3 benefits were reported as well as any other benefits with over 20% of participants reporting a benefit. For AEs, the top 3 AEs were reported and any other AEs which were reported by 15% or more of participants. These were arbitrary cut-offs to limit table entries to the most relevant symptoms; a slightly lower cut-off for AEs was chosen since they were so rare.

The top-rated treatments for each symptom were calculated by multiplying the overall net benefit by the percentage of participants who had improvements in that symptom. For each symptom, the three top-rated treatments are reported, as well as any other treatments with a score of 0.2 or higher (equivalent to 10% of participants reporting a moderate benefit).

In order to determine if any of the nutraceuticals were related to changes in ASD severity, two questions were asked on the Survey. Specifically, the ASD severity rated at 3 years of age (which would be close to most patient’s diagnosis) was compared to the currently rated ASD severity. The categories of severity were coded on a five-point scale with increasing numeric values corresponding to increasing severity. Specifically, no symptoms (0), very mild symptoms (1), mild symptoms (2), moderate symptoms (3), and severe symptoms (4). The current ASD severity was subtracted from the severity at baseline (3 years of age) such that a decrease in severity would indicate an improvement. The generalized linear model performed in IBM SPSS PASW Release 18.0.0 (Armonk, New York) was used to analyze change in severity. The model included gender (male, female), developmental profile, number of antibiotic treatments in the first 3 years of life (since that has been reported higher in ASD), and baseline ASD severity. In general, only treatments that were used by 100 respondents or more were analyzed to ensure generalizability and a wide range of ASD severity changes. Two different approaches were used. First, it was determined whether use of the nutraceutical was associated with improvements in ASD symptoms by comparing those who used the nutraceutical to those who did not. Second, the association between the perceived benefits of the nutraceutical with the change in ASD symptoms was examined by comparing current ASD severity versus severity at 3 years old. This later analysis including an interaction between treatment and severity at 3 years of age in order to determine whether the change in severity associated with the treatment was affected by the severity of ASD at age 3 years of age. A one-way analysis of variance was also used to determine whether severity at 3 years of age was related to the use of any treatment studied.

## 3. Results

### 3.1. Demographics and Medical History

The characteristics of the 1286 participants and their medical history are outlined in Table 2. The majority of the surveys were completed by the primary caregiver of an individual with ASD (85%). More than half of the surveys were for children under 13 years old (54%), with 21% for teenagers and 16% for young adults (18 years or older). Seventy six percent of participants were male, and 24% were female. Autism was the most frequent diagnosis (43%), followed by Autism Spectrum Disorder (22%) and Asperger’s syndrome (14%). The most common developmental history was “Abnormal development from early infancy, with no major regression or plateau in development” (32%). Furthermore, most participants received antibiotics during their first 3 years of life, with a median of 3 rounds. Most participants had moderate autism-related symptoms at age 3 years old (38%) and currently (38%).

### 3.2. Nutraceuticals

Of the 123 nutraceuticals included in the Survey (found in Supplemental Table S1), 58 had 20 or more responses and are reported here. These nutraceuticals are reported in eight general categories—the categories and the number of nutraceuticals for each category are: amino acids (4), essential fatty acids (7), glutathione-related nutraceuticals (4), individual minerals (9), individual vitamins/vitamin-like nutraceuticals (22), multivitamins (3), sleep treatments (3), and others (5). The most commonly used treatments were generic child/adult multivitamin (34%), melatonin (29%), omega 3 fatty acids (15%), vitamin C (14%), krill oil (13%), fish oil (13%), vitamin D (12%), magnesium (12%), Epsom salts (11%), and zinc (10%).

#### 3.2.1. Amino Acids

**Figure 1:**
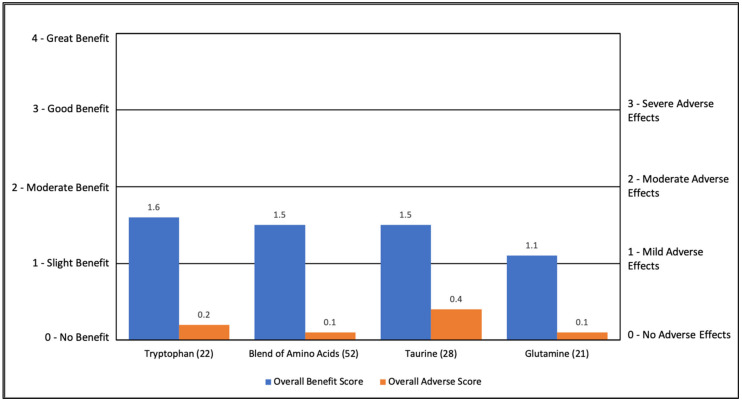
Overall Benefit Score and Adverse Effect Score for Amino Acid Treatments from Highest Overall Benefit to Lowest Overall Benefit.

Amino Acids were rated as having a slight to moderate (1.1 to 1.6) overall perceived benefit with minimal AEs (0.1 to 0.4). For the amino acid blend, glutamine and taurine, the primary benefit was general benefit (43–57%) with small benefits in other symptoms. For tryptophan, the primary benefits were helping with falling asleep and staying asleep (see Table S2 and Figure 1).

#### 3.2.2. Fatty Acids

**Figure 2:**
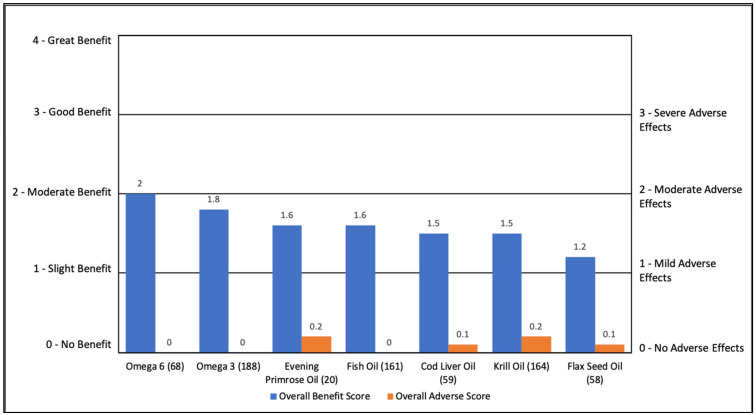
Overall Benefit Score and Adverse Effect Score for Fatty Acid Treatments from Highest Overall Benefit to Lowest Overall Benefit.

Fatty Acids (FA) were rated as having a moderate to good benefit (1.2 to 2) with minimal overall AEs (0 to 0.2). For all FAs, the primary benefit was general benefit (32% to 59%), with secondary benefits in attention and cognition. See Table S3 and Figure 2.

#### 3.2.3. Glutathione-Related Nutraceuticals

**Figure 3:**
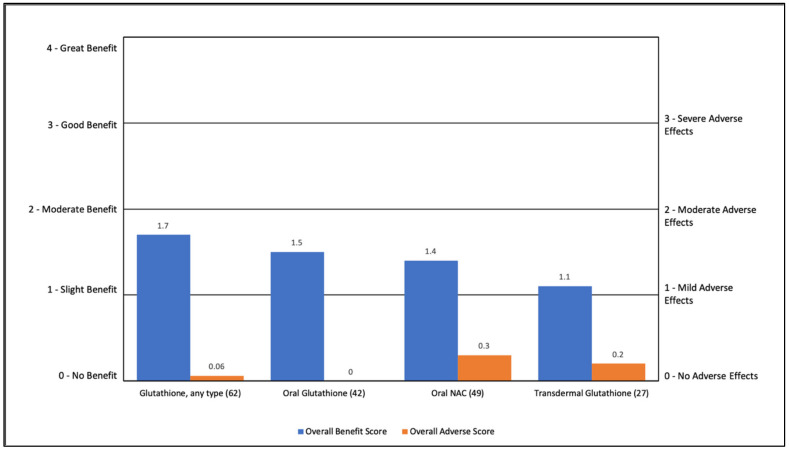
Overall Benefit Score and Adverse Effect Score for Glutathione-Related Treatments from Highest Overall Benefit to Lowest Overall Benefit.

Glutathione-related nutraceuticals (including NAC) were rated as having a slight to moderate benefit (1.1 to 1.7) with minimal AEs (0 to 0.3). The most common benefit was general benefit (4% to 56%). See Table S3 and Figure 3.

#### 3.2.4. Individual Minerals

**Figure 4:**
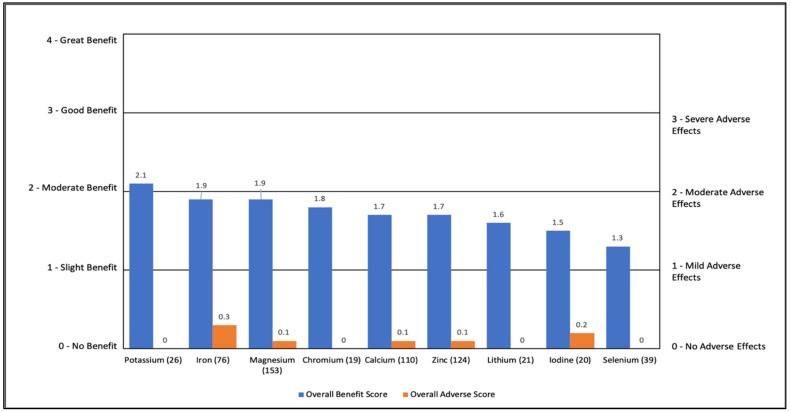
Overall Benefit Score and Adverse Event Score for Individual Minerals from Highest Overall Benefit to Lowest Overall Benefit.

Individual minerals were rated as having a slight to moderate benefit (1.3 to 2.1) with minimal AEs (0–0.3). The most common benefit was general benefit (15% to 70%). Lithium also helped with anxiety (24%), and magnesium helped with constipation (27%). Iron caused some gastrointestinal adverse effects in 17%. See Table S4 and Figure 4.

#### 3.2.5. Individual Vitamins/Vitamin-like Nutraceuticals

**Figure 5:**
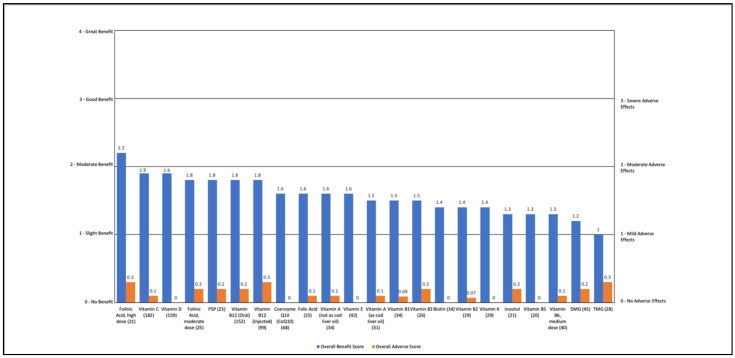
Overall Benefit Score and Adverse Event Score for Individual Vitamins/Vitamin-like Nutraceuticals from Highest Overall Benefit to Lowest Overall Benefit.

Individual vitamins and vitamin-like nutraceuticals were rated as having slight to moderate overall benefits (1.0 to 2.2) with minimal AEs (0 to 0.3). The most common benefit was general overall benefit (14% to 62%). High dose folinic acid (above 5 mg/day) improved cognition (33%), attention (29%), and language/communication (24%). Moderate dose folinic acid (below 5 mg/day) also improved language/communication (20%). P5P improved anxiety (20%) and TMG improved language/communication (29%). Injected vitamin B12 improved language/communication (30%), cognition (28%), and attention (20%). Oral vitamin B12 improved cognition (25%) and language/communication (18%). Vitamin C also improved overall health (27%). See Table S6 and Figure 5.

#### 3.2.6. Multivitamins

**Figure 6:**
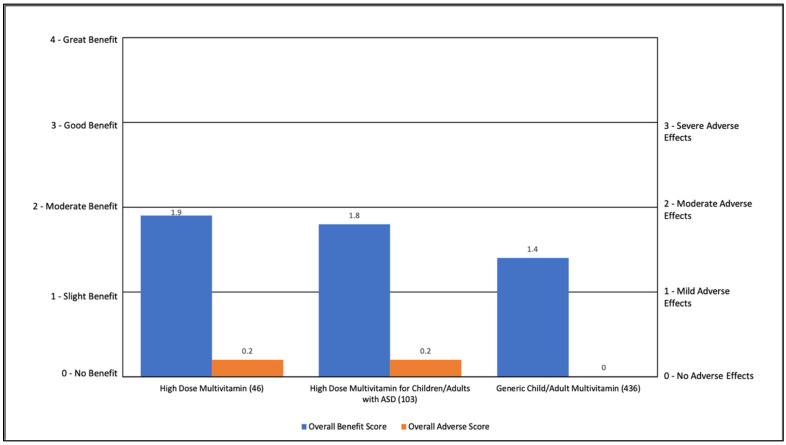
Overall Benefit Score and Adverse Event Score for Multivitamins from Highest Overall Benefit to Lowest Overall Benefit.

Multivitamins were rated as having a slight to moderate benefit (1.4 to 1.9) with minimal AEs (0.0 to 0.2). The most common benefit was general benefit (50–55%). High dose multivitamin also improved general health (26%), and a high dose multivitamin, specifically designed for ASD, improved cognition (21%). See Table S7 and Figure 6.

#### 3.2.7. Sleep-Related Nutraceuticals

**Figure 7:**
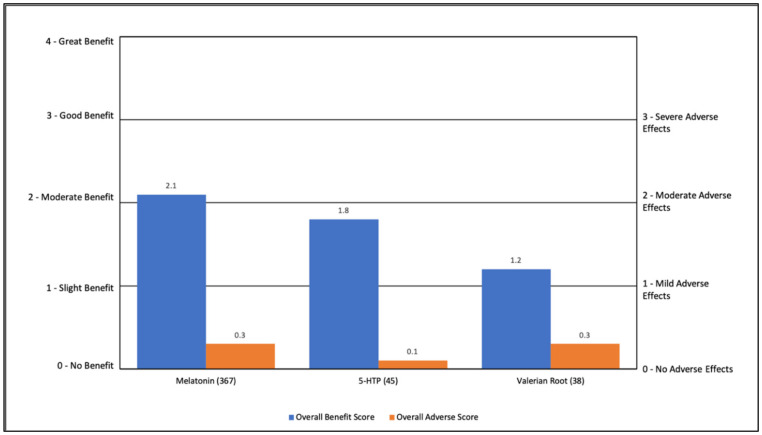
Overall Benefit Score and Adverse Event Score for Sleep Treatments from Highest Overall Benefit to Lowest Overall Benefit.

Sleep-related nutraceuticals were rated as having slight to moderate benefit (1.2–2.1), with minimal AEs (0.1 to 0.3). The primary benefit was falling asleep (36–74%), followed by staying asleep (27–35%). For 5-HTP, there was also a general benefit (27%). See Table S8 and Figure 7. It is noteworthy that melatonin had the highest overall benefit score and was used by a very high number of participants.

#### 3.2.8. Other Miscellaneous Nutraceuticals

**Figure 8:**
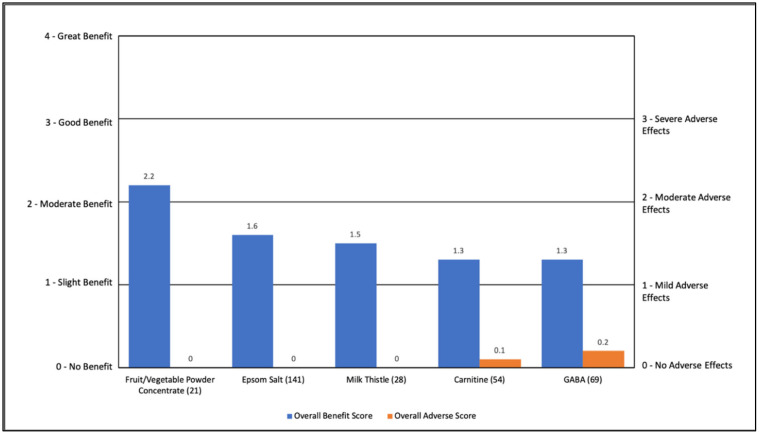
Overall Benefit Score and Adverse Effect Score for Other Miscellaneous Nutraceuticals from Highest Overall Benefit to Lowest Overall Benefit.

For other miscellaneous nutraceuticals, the general benefit ranged from 1.3 to 2.2 (slight to moderate benefit) with minimal AEs (0.0 to 0.2). All of these nutraceuticals had improvements in general benefit (22% to 67%). Epsom salts improved aggression/agitation (35%) and attention (26%). A fruit/vegetable powder concentrate also improved constipation (24%) and general health (24%). GABA improved anxiety (26%). See Table S9 and Figure 8.

#### 3.2.9. Average of All Nutraceuticals

Averaging all the nutraceuticals reported in this paper, the average Overall Benefit and Overall AE was 1.6 (SD = 0.3) and 0.1 (SD = 0.1), respectively, reflecting that participants reported on average slight to moderate benefits with minimal adverse effects.

#### 3.2.10. Top Nutraceuticals by Symptom

Table 3 presents the top-rated nutraceuticals for 24 different symptoms. For most symptoms, nutraceuticals were moderately effective (net benefit scores >0.25), including aggression/agitation, anxiety, attention, cognition, constipation, diarrhea, general benefit, health, hyperactivity, irritability, language/communication, falling asleep, staying asleep, and social interaction/understanding. Other symptoms were only slightly affected (net benefit scores between 0.10 and 0.25) such as depression, eczema/skin problems, lethargy, obsessive-compulsive symptoms, reflux/vomiting, sensory sensitivity, stimming and tics/involuntary movements (Table 3).

It is important to note that less common problems, such as seizures, might receive lower scores since fewer individuals have these problems. These ratings should be interpreted cautiously, as they are averages, but they suggest which treatments families sensed were most helpful for a given symptom, which can potentially help guide treatment selection and future research.

#### 3.2.11. Overall Effects of Nutraceuticals

As a final part of this Survey, participants were asked to rate the overall effect of nutraceuticals (Table 4). A total of 77% of participants reported that nutraceuticals had a positive effect, ranging from slightly better (24%) to much better (27%), with 23% reporting no effect, and no reports that they generally resulted in worsened symptoms.

### 3.3. Analysis of the Effect on Specific Supplements on Change in Severity

To study the change in ASD severity related to nutraceuticals, nutraceuticals with at least 100 responses were selected in order to ensure there were enough cases to provide an adequate range of change in ASD severity. Since there were multiple categories of Omega 3 fatty acids (Fish Oils, Omega 3 Fatty Acids, Krill Oil) and B12 (oral and injected) these nutraceuticals were combined into categories. Thus, nutraceuticals selected included B12 (*n* = 170), Omega 3 fatty acids (*n* = 276), Epsom salt baths (*n* = 141), calcium (*n* = 110), magnesium (*n* = 153), zinc (*n* = 124), Vitamin C (*n* = 182), Vitamin D (*n* = 159), generic multivitamin (MVI) (*n* = 436), autism specific MVI (*n* = 103), and melatonin (*n* = 367). Because two other MVIs were being studied, high dose MVI (*n* = 45) was also included in the analysis. Because of the interest in the difference between injected vs. oral B12, the analysis was conducted on the separate groups of oral B12 (*n* = 127) and injected B12 (*n* = 76) as well as any B12 use. The analysis adjusted for baseline severity at age 3 years of age, developmental profile, number of rounds of antibiotic used in infancy, and gender.

#### 3.3.1. Specific Nutraceutical Use

**Figure 9:**
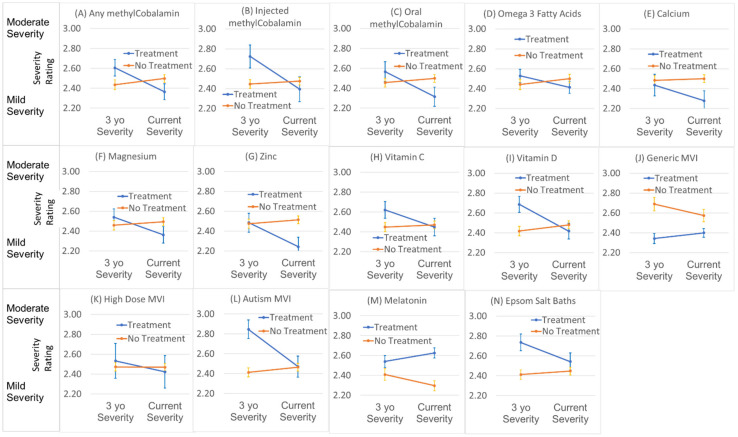
Relationship between nutraceuticals and change in autism severity from 3 years of age to the current age.

First, the analysis determined whether the changes in severity from 3 years of age to the current age was related to taking a nutraceutical regardless of the reported specific beneficial response. The uses of any B12 [χ(1)^2^ = 11.79, *p* < 0.001], injected B12 [χ(1)^2^ = 5.58, *p* = 0.01] or oral B12 [χ(1)^2^ = 11.48, *p* = 0.001], Calcium [χ(1)^2^ = 8.29, *p* < 0.01], Magnesium [χ(1)^2^ = 5.83 *p* = 0.01], Zinc [χ(1)^2^ = 20.46 *p* < 0.001], Vitamin D [χ(1)^2^ = 6.66 *p* = 0.01], or a multivitamin specifically formulated for ASD [χ(1)^2^ =7.00 *p* < 0.01] were significantly related to a positive improvement in ASD symptoms (a reduction in ASD severity) as seen in Figure 9.

The change in ASD severity was also related to baseline severity at 3 years of age in all of the analyses, which included taking B12 [χ(1)^2^ = 336, *p* < 0.001], B12 injections [χ(1)^2^ = 332, *p* < 0.001], oral B12 [χ(1)^2^ = 341, *p* < 0.001], Omega 3 Fatty Acids [χ(1)^2^ = 336, *p* < 0.001], Epsom Salt Baths [χ(1)^2^ = 329, *p* < 0.001], Calcium [χ(1)^2^ = 343, *p* < 0.001], Magnesium [χ(1)^2^ = 338, *p* < 0.001], Zinc [χ(1)^2^ = 350, *p* < 0.001], Vitamin C [χ(1)^2^ = 335, *p* < 0.001], Vitamin D [χ(1)^2^ = 327, *p* < 0.001], Generic MVI [χ(1)^2^ = 327, *p* < 0.001], high dose MVI [χ(1)^2^ = 337, *p* < 0.001], autism specific MVI [χ(1)^2^ = 321, *p* < 0.001], and Melatonin [χ(1)^2^ = 355, *p* < 0.001]. In all models, a higher baseline severity was associated with a larger positive change in development as might be expected as higher severity patients have more potential for improvements.

Almost all of the associations shown in Figure 9 demonstrate that treatment was associated with greater improvements. The exceptions were generic multi-vitamin, presumably because that meant participants did not take a multi-vitamin specific for ASD, and melatonin, probably because it treats a specific problem and is given to children with sleep disorders who may require additional non-nutraceutical treatments.

#### 3.3.2. Perceived Benefit and Change in Autism Severity

**Figure 10:**
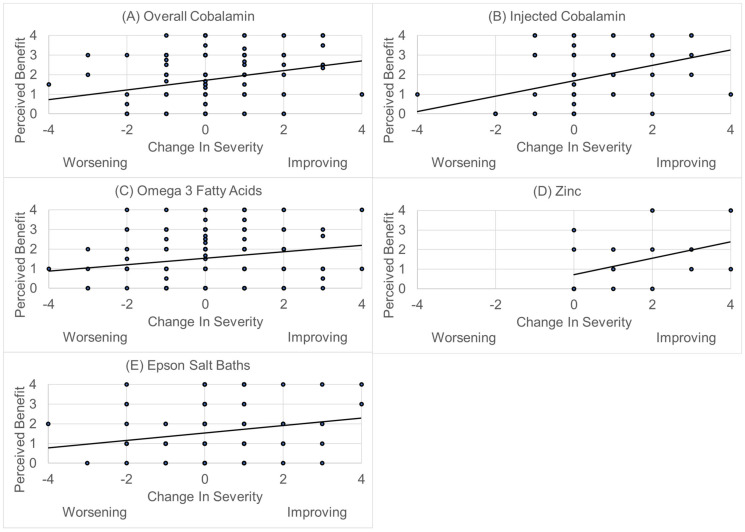
Association between change in ASD severity with the perceived benefit of the nutraceutical (**A**) Overall methylcobalamin; (**B**) Injected methylcobalamin; (**C**) Omega 3 Fatty Acids; (**D**) Zinc; (**E**) Epsom Salt Baths.

A positive change in ASD severity was associated with the perceived benefit of any B12 supplement [χ(1)^2^ = 10.14, *p* = 0.001] and the baseline ASD severity [χ(1)^2^ = 94.85, *p* < 0.001] (Figure 10A), as well as the perceived benefit of injected B12 supplement [χ(1)^2^ = 27.45, *p* < 0.001] and the baseline ASD severity [χ(1)^2^ = 61.34, *p* < 0.001] (Figure 10B). Interestingly, the pattern of the child’s development also affected the change in ASD severity when controlling for the benefit of injected B12 [χ(1)^2^ = 24.32, *p* < 0.001]. This was due to the children with early onset ASD demonstrating significantly greater benefit (1.5) as compared to those who had a clinical regression and then a developmental plateau (−0.31), those with only a plateau (0.69) or those with a major developmental regression (0.46) when controlling for the perceived benefit of B12 injections.

A positive change in ASD severity was associated with the perceived benefit for Omega 3 Fatty Acids [χ(1)^2^ = 6.10, *p* = 0.01] and the baseline ASD severity [χ(1)^2^ = 148.38, *p* < 0.001] (Figure 10C). A positive change in ASD severity was associated with the perceived benefit in zinc supplementation [χ(1)^2^ = 7.25, *p* < 0.01] and the baseline ASD severity [χ(1)^2^ = 86.29, *p* < 0.001]. However, the effect of baseline ASD severity resulted in the perceived benefit only being obvious in the most severely affected patients (Figure 10D). Finally, a positive change in ASD severity was associated with the perceived benefit in Epsom salts [χ(1)^2^ = 6.59, *p* = 0.01] and the baseline ASD severity [χ(1)^2^ = 66.80, *p* < 0.001] (Figure 10E).

We also compared whether the severity of the diagnosis was related to starting any supplement. Those that took injected B12 [F(1710) = 4.244, *p* = 0.04], Epsom salt baths [F(1710) = 9.630, *p* < 0.01], Vitamin D [F(1710) = 7.184, *p* < 0.01], or MVI specific for ASD [F(1710) = 13.752, *p* < 0.001] had a higher severity at age 3 years of age whereas those that took a standard MVI [F(1710) = 16.640, *p* < 0.001] had a lower severity at age 3 years of age. The interaction with severity at 3 years of age and treatment was included in the linear model to determine if this effected the change in severity with treatment. For injected B12 [χ(1)^2^ = 7.77, *p* < 0.01], Oral B12 [χ(1)^2^ = 3.71, *p* = 0.05], Epsom salt baths [χ(1)^2^ = 3.70, *p* = 0.05], Calcium [χ(1)^2^ = 4.56, *p* < 0.05], Magnesium [χ(1)^2^ = 3.93, *p* < 0.05], and Zinc [χ(1)^2^ = 13.16, *p* < 0.001], the severity of autism at age 3 affected response to the treatment such that more severe individuals demonstrated a slightly lower response to some treatments.

## 4. Discussion

This study presents the Survey results of participants’ reports of the perceived effectiveness and potential AEs of a wide range of nutraceuticals used in individuals with ASD. Nutraceuticals were generally reported to have a higher benefit compared to their AEs, with an average of 1.6 (slight/moderate benefit) and 0.1 (minimal AE), respectively. Reported benefits were generally in the slight/moderate range, and AEs were minimal.

The results of this study found significant benefits for many nutraceuticals with minimal adverse effects and are consistent with the findings of a number of clinical trials studying nutraceuticals in ASD. For example, double-blind, placebo-controlled studies, and/or meta-analyses have reported improvements in children with ASD using L-carnitine [32,33], Coenzyme Q10 (ubiquinone) [34], digestive enzymes [35,36], high dose folinic acid (1–2 mg/kg/day) [24,25,26], MB12 injections [37], melatonin [9,38,39,40,41,42], a multivitamin/mineral supplement designed specifically for ASD [26,27], NAC [29,43,44,45], omega 3 fatty acids [46,47], vitamin C [48], vitamin D3 [49,50], and possibly B6/Mg [51,52]. Open-label studies in ASD have also reported benefits for B vitamins [53,54], biotin [55], folic acid [56], an herbal formula [57], glutathione [58], iron [59], vitamin A [60] and zinc [61,62].

Some of the nutraceuticals in this Survey have not been previously studied in ASD including an amino acid blend, glutamine, taurine, tryptophan, evening primrose oil, flax seed oil, krill oil, calcium, chromium, iodine, lithium, potassium, selenium, vitamin E, vitamin K, valerian root, Epsom salts, GABA, and milk thistle. Thus, this Survey provides preliminary data on the effects (both beneficial and adverse) of these unstudied treatments which can help guide researchers to choose the most promising treatments to study in the future.

Some of the treatments reviewed may not only help certain symptoms of ASD but also treat underlying metabolic abnormalities associated with ASD. For example, mitochondrial dysfunction is relatively common in individuals with ASD [10,63] and is potentially treated with carnitine, Coenzyme Q10, B vitamins, and vitamin C [64]. Oxidative stress is also commonly associated with ASD [13] and is potentially treatable with antioxidants such as folinic acid, MB12, vitamin C, vitamin E, glutathione, ribose, and NADH. Melatonin is also an antioxidant and has positive effects on mitochondrial function [65].

Furthermore, children with ASD have been found to have multiple abnormalities related to one-carbon metabolism, including lower plasma levels of methionine, S-adenosylhomocysteine (SAM), homocysteine, cystathionine, cysteine, and total glutathione (GSH), as well as significantly higher concentrations of S-adenosylhomocysteine (SAH), adenosine, and oxidized glutathione (GSSG) [7,8]. Some studies have demonstrated that many children with ASD have a partial blockage in the transportation of folates into the brain due to an autoantibody to the folate receptor alpha, the primary mechanism which transports folate across the blood-brain barrier [66,67]. High dose folinic acid (1–2 mg/kg/day) has been shown to be an effective treatment for children with ASD with primary improvements in language in a double-blind placebo-controlled study [24], consistent with the findings of this Survey. Also consistent with this Survey, an open-label study found that high-dose folinic acid is effective for improving attention in children with ASD who possess the folate receptor alpha antibody [66], and two other placebo-controlled studies have also reported improvements with folinic acid in ASD [25,26]. These positive studies on the benefits of folinic acid are consistent with the results of Table 3, which demonstrates that folinic acid and vitamin B12 are two of the top-rated treatments for many ASD-related symptoms.

These abnormalities in one-carbon metabolism often result in problems in methylation and transsulfuration in ASD, resulting in a reduction in the production of glutathione [68]. In fact, these abnormalities appear to be so prevalent that they may be diagnostic for ASD [69]. Several studies [70,71] have addressed treatment of these linked pathways by providing cobalamin and folate derivatives to supplement the linked methylation-folate pathway in order to enhance the production of glutathione, while other studies have supplemented glutathione directly [58]. The findings of these studies of the benefits of cobalamin, folate, and glutathione are consistent with the results of this Survey.

It is interesting to compare the results of this Survey for nutraceuticals versus the results of this Survey for pharmaceuticals reported previously [31]. Averaging all nutraceuticals and all pharmaceuticals, the nutraceuticals had significantly higher Overall Benefit (1.59 vs. 1.39, *p* = 0.01) and significantly lower Overall Adverse Effect (0.1 vs. 0.9, *p* < 0.0001), based on a 2-sided *t*-test of the medications that had 20 or more responses [31]. Caution is needed in interpreting these results, since there are substantial variations in ratings for individual treatments. However, in general, these findings suggest that nutraceuticals may be important treatment options for ASD, and more research into nutraceuticals and how they affect metabolism is warranted.

### 4.1. Strengths of This Study

One strength of this study is that some of these nutraceuticals have not been formally studied to date; therefore, this is the first data available on these treatments for ASD. Another advantage is that a uniform rating scale was used for all treatments, so that direct comparisons between different treatments could be made—this is often not possible for comparing data from clinical trials, since different assessment tools are typically used. Finally, another strength is the large number of participants in this study.

### 4.2. Limitations of This Study

There are several limitations of this Survey to consider. One limitation is that it is based on survey data, so there may be a significant placebo effect, especially since one of the most common benefits reported was “general benefit—no specific symptom”. The ratings are based on perceived benefit (primarily by caregivers) and not by medical professionals. Age at which treatment was administered was not collected, which is a limitation of the study. Furthermore, there was no data collected on the dosages or durations of treatments (other than high versus low dose folinic acid). Therefore, various doses and durations of treatments may have been used by participants. Another limitation is the ASD-related diagnoses were not confirmed with standardized testing but were gathered by participant self-report. Finally, there is the potential for recall bias, where participants may not completely remember the effects of certain treatments. This may be reflected by the fact that no participants listed any of the nutraceuticals as causing worsening in ASD-related symptoms.

## 5. Conclusions

This Survey provides important information on the overall and specific benefits and adverse effects of 58 of the most commonly used nutraceuticals in ASD. The Overall Benefits were rated slightly higher for the nutraceuticals than for the most commonly used pharmaceuticals reported in the previous paper, with significantly lower ratings of adverse effects. The perception of participants of slight/moderate benefit with minimal adverse effects potentially explains why nutraceuticals were used by 75.2% of individuals with ASD in the Survey. This is consistent with the growing number of positive randomized clinical trials of nutraceuticals in ASD. Further research into nutraceutical treatments for treating biochemical differences and ASD symptoms is warranted.

## Figures and Tables

**Table 1:** All Symptom Options.

Benefited Symptom Options	Adverse Symptom Options
General benefit, no one particular symptom	General worsening, no one specific symptom
Aggression/Agitation	Aggression/Agitation
Anxiety	Anxiety
Attention	Bedwetting/Bladder Control
Cognition (ability to think)	Behavior problems
Constipation	Decreased cognition (difficulty thinking/remembering)
Depression	Depression
Diarrhea	Dizziness/Unsteadiness
Eczema/Skin problem	Dry mouth
Health (fewer illnesses and/or less severe illnesses)	Fatigue/Drowsiness
Hyperactivity	Gastrointestinal problems
Irritability	Headache/Migraine
Language/Communication	Hyperactivity
Lethargy (easily tired)	Irritability
OCD	Liver/Kidney problem
Reflux/Vomiting	Loss of appetite
Seizures	Nausea
Self-Injury	Rash
Sensory Sensitivity	Seizures
Sleep (falling asleep)	Self-injury
Sleep (staying asleep)	Sleep Problems
Social Interaction and Understanding	Stimming/Perseveration/Desire for Sameness
Stimming/Perseveration/Desire for Sameness	Tics/Abnormal movements
Tics/Abnormal movements	Weight gain
	Weight loss

**Table 2:** Completion, Age, Gender, Medical Diagnosis, Developmental History, and Antibiotic Use.

	N	%
**Survey Completed By**		
Primary Caregiver of an Individual with Autism	1094	85%
Adult with High-Functioning Autism 18 years or older who does not have a guardianship	89	7%
Adult with Autism with their Mother/Father/Childhood Guardian	39	3%
Grandparent of an Individual with Autism ^1^	58	5%
Other	6	0%
**Age of Participant**		
Child (under 13 years old)	692	54%
Teenager (13–17 years old)	274	21%
Young Adult (18–30 years old)	202	16%
Adult (over 30 years old)	116	9%
**Gender of Participant**		
Male	973	76%
Female	305	24%
Other	5	0%
**Current Medical Diagnosis**		
Autism	548	43%
Asperger’s Syndrome	186	14%
Autism Spectrum Disorder	289	22%
High-Functioning Autism	148	12%
Pervasive Developmental Disorder—Not Otherwise Specified (PDD-NOS)	76	6%
No current diagnosis, but he/she was on the autism spectrum previously	18	1%
Other	21	2%
**Developmental History**		
Abnormal development from early infancy, with no major regression or plateau in development	410	32%
Normal development, followed by a plateau in development that lasted for several months or longer	284	22%
Normal development, followed by major regression	278	22%
Normal development, followed by a major regression and a plateau lasting several months or longer	168	13%
Other	127	10%
**Number of Rounds of Antibiotics during the first three years of life**		
Average	9.3	
Median	3.0	
None	148	14%
1 Round	183	17%
2 Rounds	138	13%
3 Rounds	163	15%
4 Rounds	67	6%
5 Rounds	63	6%
6–7 Rounds	79	7%
8–10 Rounds	90	8%
11–15 Rounds	35	3%
16–20 Rounds	22	2%
21+ Rounds	84	8%
**Severity of autism-related symptoms at age 3**		
No autistic symptoms	50	4%
Nearly normal, with only very mild symptoms	227	18%
Mild autism	290	23%
Moderate autism	474	38%
Severe autism	222	18%
**Severity of autism-related symptoms currently**		
No autistic symptoms	14	1%
Nearly normal, with only very mild symptoms	200	16%
Mild autism	390	31%
Moderate autism	475	38%
Severe autism	186	15%

^1^. Grandparents were taken from those responded with “other” and noted they were grandparents. Numbers may not add up to 100% due to rounding.

**Table 3:** Top Nutraceuticals for Symptoms from Highest Overall Net Benefit Rating to Lowest Overall Net Benefit Rating.

Symptoms	Nutraceuticals (Overall Net Benefit Rating)
General benefit, no one particular symptom	Fruit/Vegetable Powder Concentrate (1.49), Potassium (1.30), Omega 6 (1.18), Vitamin C (1.05), Vitamin D (1.00), CoQ10 (1.00), High Dose Folinic Acid (1.00), Chromium (0.97), Vitamin E (0.95), Iodine (0.95), General Glutathione (0.92), Moderate Dose Folinic acid (0.92), High Dose Multivitamin (0.91), Folic Acid (0.89), Vitamin B1 (0.85), Vitamin A (not as cod liver oil) (0.85), High Dose Multivitamin for Children/Adults with ASD (0.83), Vitamin B2 (0.81), Vitamin B3 (0.78), Blend of Amino Acids (0.77), Calcium (0.76), Vitamin A (as cod liver oil) (0.75), Zinc (0.74), Generic Child/Adult Multivitamin (0.75), Omega 3 (0.74), Selenium (0.74), Vitamin B5 (0.72), Iron (0.69), Milk Thistle (0.66), Magnesium (0.66), Krill Oil (0.66), Vitamin K (0.65), PSP (0.64), Oral Vitamin B12 (0.64), Evening Primrose Oil (0.63), Oral Glutathione (0.62), Lithium (0.60), Biotin (0.60), Cod Liver Oil (0.55), Flax Seed Oil (0.52), Medium Dose Vitamin B6 (0.52), Oral NAC (0.51), Fish Oil (0.49), Carnitine (0.49), Injected Vitamin B12 (0.48), Taurine (0.47), 5-HTP (0.46), Transdermal Glutathione (0.44), Epsom Salt (0.34), DMG (0.30), GABA (0.25)
Sleep (falling asleep)	Melatonin (1.33), 5-HTP (0.61), Tryptophan (0.45), Valerian Root (0.44), Magnesium (0.21)
Cognition	High Dose Folinic Acid (0.63), Omega 6 (0.53), Cod Liver Oil (0.43), Omega 3 (0.43), Injected Vitamin B12 (0.42), Oral Vitamin B12 (0.40), Fish Oil (0.39), High Dose Multivitamin for Children/Adults with ASD (0.35), CoQ10 (0.26), High Dose Multivitamin (0.23), Lithium (0.22), Krill Oil (0.21), Moderate Dose Folinic Acid (0.20)
Sleep (staying asleep)	Melatonin (0.63), 5-HTP (0.46), Tryptophan (0.32), Valerian Root (0.24)
Aggression/Agitation	Epsom Salt (0.54), Lithium (0.22), Magnesium (0.20)
Attention	High Dose Folinic Acid (0.54), Omega 6 (0.50), Epsom Salt (0.41), High Dose Multivitamin for Children/Adults with ASD (0.32), Cod Liver Oil (0.30), Omega 3 (0.29), Evening Primrose Oil (0.28), PSP (0.26), Oral Vitamin B12 (0.26), Fish Oil (0.21)
Language/Communication	High Dose Folinic Acid (0.45), Injected Vitamin B12 (0.45), Moderate Dose Folinic Acid (0.33), Omega 6 (0.32), General Glutathione (0.32), Oral Vitamin B12 (0.30), Cod Liver Oil (0.25), Omega 3 (0.25), Epsom Salt (0.24), High Dose Multivitamin (0.23), High Dose Multivitamin for Children/Adults with ASD (0.22), Fish Oil (0.21), TMG (0.20)
Constipation	Fruit/Vegetable Powder Concentrate (0.53), Magnesium (0.48), Vitamin C (0.22)
Health (fewer illnesses and/or less severe illnesses)	Fruit/Vegetable Powder Concentrate (0.53), Vitamin C (0.51), High Dose Multivitamin (0.45), Vitamin D (0.34), Potassium (0.33), Zinc (0.31), High Dose Multivitamin for Children/Adults with ASD (0.29), Vitamin E (0.23)
Anxiety	Lithium (0.37), Magnesium (0.33), PSP (0.32), GABA (0.27), Tryptophan (0.26), High Dose Multivitamin for Children/Adults with ASD (0.22)
Social Interaction and Understanding	High Dose Folinic Acid (0.36), General Glutathione (0.26), Moderate Dose Folinic Acid (0.26), High Dose Multivitamin for Children/Adults with ASD (0.26), Injected Vitamin B12 (0.26), High Dose Multivitamin (0.23), Oral Vitamin B12 (0.22)
Irritability	High Dose Folinic Acid (0.27), Magnesium (0.26), Epsom Salt (0.23), High Dose Multivitamin for Children/Adults with ASD (0.22)
Sensory Sensitivity	High Dose Multivitamin for Children/Adults with ASD (0.22), Lithium (0.15), Injected Vitamin B12 (0.12)
Lethargy (easily tired)	Carnitine (0.21), Chromium (0.19), Injected Vitamin B12 (0.15)
Depression	Epsom Salt (0.18), Tryptophan (0.13), Vitamin D (0.11)
Hyperactivity	High Dose Folinic Acid (0.18), High Dose Multivitamin for Children/Adults with ASD (0.13), Magnesium (0.12)
Stimming/Perseveration/Desire for Sameness	Evening Primrose Oil (0.14), High Dose Multivitamin for Children/Adults with ASD (0.13), Tryptophan (0.13)
OCD	PSP (0.13), Vitamin B3 (0.10), High Dose Folinic Acid (0.09)
Tics/involuntary movements	Tryptophan (0.13), Potassium (0.08), High Dose Multivitamin (0.08)
Eczema/Skin problems	Biotin (0.12), Vitamin E (0.08), Vitamin D (0.07)
Diarrhea	Fruit/Vegetable Powder Concentrate (0.11), Glutamine (0.08), High Dose Multivitamin for Children/Adults with ASD (0.06)
Seizures	High Dose Folinic Acid (0.09), Moderate Dose Folinic Acid (0.07), Oral Vitamin B12 (0.01)
Self-Injurious behaviors	High Dose Folinic Acid (0.09), Oral Glutathione (0.07), High Dose Multivitamin for Children/Adults with ASD (0.06)
Reflux/Vomiting	Epsom Salt (0.06), Milk Thistle (0.05), Vitamin E (0.04)

**Table 4:** Rating of the Overall Effects of Nutraceuticals.

Percentage of Responses	
Much Better (3)	27%
Somewhat Better (2)	26%
Slightly Better (1)	24%
No Effect (0)	23%
Mildly Worse (−1)	0%
Somewhat Worse (−2)	0%
Much Worse (−3)	0%
Average	1.6

## Data Availability

The data presented in this study are available on request from the corresponding author. The data are not publicly available due to plans for additional analysis.

## Supplementary Materials

The following are available online at https://www.mdpi.com/article/10.3390/jpm11090878/s1, Table S1. List of All Nutraceuticals in Survey. Table S2. Amino Acids. Table S3. Fatty Acids. Table S4. Glutathione-related Nutraceuticals. Table S5. Individual Minerals. Table S6. Individual Vitamins/Vitamin-like Nutraceuticals. Table S7. Multivitamins. Table S8. Sleep Treatments. Table S9. Other Miscellaneous Nutraceuticals.
