# Supplementary material for: Ratings of the Effectiveness of Nutraceuticals for Autism Spectrum Disorders: Results of a National Survey
Source: J Pers Med. 2021 Aug 31;11(9):878. doi: 10.3390/jpm11090878 (PMC8470413; doi:10.3390/jpm11090878)
Supplement: Supplementary file 1 [file jpm-11-00878-s001.zip › jpm-1336889-supplementary.pdf]

Table S1. List of All Nutraceuticals in Survey

Nutritional Supplements
5-HTP
Alanine
Aloe or Super Aloe
Alpha Ketoglutaric Acid
Amino Acid Complex
Arginine
Asparagine
Aspartic Acid
Biotin
Black Currant Seed Oil
Blend of Amino Acids
Borage Oil
Calcium
Caprylic acid
Carnitine
Carnosine
Chia Seed
Choline
Chromium
Citrus seed extract
Cod Liver oil
Copper
CoQ10
Cysteine
Digestive Enzymes
DMG
Evening Primrose Oil
Fish Oil (not from liver)
Flax Seed Oil
Flax Seeds

Folic Acid
Folinic Acid
Folinic Acid, High dose (over 5 mg/day)
Folinic Acid, Moderate dose (under 5 mg/day)
Fruit/ Vegetable Powder Concentrate
GABA
Generic child/adult multivitamin
Ginkgo Bilboa
Glutamic Acid
Glutamine
Glutathione
Glutathione -- IV
Glutathione -- Oral
Glutathione -- Transdermal
Glycine
Herbal
High-dose multivitamin
High-dose multivitamin designed for children/adults with ASD
Histidine
Inositol
Iodine
Iron
Isoleucine
Krill Oil
Lactulose
Lamisil
Leucine
Lithium (as nutritional supplement)
Magnesium
Magnesium Citrate
Manganese
Melatonin
Methionine

Methylcobalamin (Methyl B12)
Methylcobalamin Injections
Milk Thistle (Silymarin) for Liver Function
Mineral oil
Miralax
Molybdenum
NAC (N-acetylcysteine) -- Oral
NAC (N-acetylcysteine) -- Transdermal
Olive Leaf Extract
Omega-3
Omega-6
Oregano concentrate
P5P
Pau d’arco
Phenylalanine
Phosphorus
Piracetam
Potassium
Pregnenolone
Probiotics
Proline
Pycnogenol
Ribose
Saccharomyces boulardii
Saline Enemas
SAMe
Selenium
Slippery Elm
Sporanox
St. John's Wort
Sulforaphane
Sulfur as Chondroitin Sulfate
Sulfur as Epsom Salts in a bath

Sulfur as Epsom Salts in a lotion
Sulfur as Glucosamine Sulfate
Sulfur as MSM
Taurine
Threonine
TMG
Tryptophan
Tyrosine
Undecylenic acid
Valerian Root
Valine
Vitamin A (as cod liver oil)
Vitamin A (not as cod liver oil)
Vitamin B1 (thiamin)
Vitamin B12 (cobalamin) -- Injection
Vitamin B12 (cobalamin) -- Oral
Vitamin B2 (riboflavin)
Vitamin B3 (as NADH)
Vitamin B3 (niacin/niacinamide)
Vitamin B5 (pantethine/pantothenic acid)
Vitamin B6 (pyridoxine), High dose (over 100 mg/day)
Vitamin B6 (pyridoxine), Moderate dose (under 100 mg/day)
Vitamin C
Vitamin D
Vitamin E
Vitamin K
Zinc

Table S2. Amino Acids. The table lists the Overall Benefit Score, the most common benefits, the Overall Adverse Score, and the most common adverse effects.

**Amino Acids**

	Treatments (n)			
	**Tryptophan (22)**	**Blend of Amino Acids (52)**	**Taurine (28)**	**Glutamine (21)**
**Overall Benefit Score**	1.6	1.5	1.5	1.1
**Symptoms-Benefit**				
General benefit, no one particular symptom		54%	43%	57%
Aggression/Agitation			7%	
Anxiety	18%	12%	14%	
Attention		13%		10%
Diarrhea				10%
Falling Asleep	32%			
Staying Asleep	23%			
**Overall Adverse Score**	0.2	0.1	0.4	0.1
**Symptoms-Adverse**				
General worsening, no one specific symptom	5%	4%		
Anxiety	9%		7%	
Aggression/Agitation	5%		7%	5%
Bedwetting/Bladder control problem		2%		
Behavioral Problems				5%
Hyperactivity		2%		10%

Irritability			7%	

Table S3. Fatty Acids. The table lists the Overall Benefit Score, the most common benefits, the Overall Adverse Score, and the most common adverse effects.

	**Fatty Acids**						
	**Treatments (n)**						
	**Omega 6 (68)**	**Omega 3 (188)**	**Evening Primrose Oil (20)**	**Fish Oil (161)**	**Cod Liver Oil (59)**	**Krill Oil (164)**	**Flax Seed Oil (58)**
**Overall Benefit Score**	2	1.8	1.6	1.6	1.5	1.5	1.2
**Symptoms-Benefit**							
General Benefit, no one particular symptom	59%	43%	45%	32%	40%	53%	47%
Attention	25%	17%	20%	14%	22%		3%
Cognition	26%	25%	10%	25%	32%	17%	
Constipation							16%
Language/Communication						10%	
**Overall Adverse Score**	0	0	0.2	0	0.1	0.2	0.1
**Symptoms-Adverse**							
General worsening, no one specific symptom		1%					
Aggression/Agitation		1%	10%		1%	3%	
Anxiety		1%	5%				
Gastrointestinal problems				1%	1%	3%	3%
Hyperactivity			5%		2%		

Rash							2%
Seizures						3%	
Stimming/Perseveration/Desire for Sameness				1%			

Table S4. Glutathione-related Nutraceuticals. The table lists the Overall Benefit Score, the most common benefits, the Overall Adverse Score, and the most common adverse effects.

	**Glutathione-related Nutraceuticals**			
	**Treatments (n)**			
	**Glutathione, any type (62)**	**Oral Glutathione (42)**	**Oral NAC (49)**	**Transdermal Glutathione (27)**
**Overall Benefit Score**	1.7	1.5	1.4	1.1
**Symptoms-Benefit**				
General Benefit, no one particular symptom	56%	43%	4%	41%
Attention			4%	4%
Cognition		12%	4%	4%
Constipation				
Language/Communication	19%			
Social Interaction and Understanding	16%	7%		
**Overall Adverse Score**	0.06	0	0.3	0.2
**Symptoms-Adverse**				
General worsening, no one specific symptom	3%			4%
Aggression/Agitation			4%	
Anxiety			4%	
Behavioral problems			4%	
Hyperactivity				
Irritability	3%	2%		

Rash				4%
Stimming/Perseveration/Desire for Sameness	2%			

Table S5. Individual Minerals. The table lists the Overall Benefit Score, the most common benefits, the Overall Adverse Score, and the most common adverse effects.

	Individual Minerals								
	Treatments (n)								
	**Potassium (26)**	**Iron (76)**	**Magnesium (153)**	**Chromium (19)**	**Calcium (110)**	**Zinc (124)**	**Lithium (21)**	**Iodine (20)**	**Selenium (39)**
**Overall Benefit Score**	2.1	1.9	1.9	1.8	1.7	1.7	1.6	1.5	1.3
**Symptoms-Benefit**									
General Benefit, no one particular symptom	62%	43%	37%	53%	50%	47%	38%	70%	56%
Aggression/Agitation							14%		
Anxiety			18%		5%		24%	5%	
Cognition				11%				5%	
Constipation			27%						3%
Falling Asleep					4%				
Health	15%	7%				19%			
Irritability						5%			
Lethargy	8%	9%		11%					
Sensory Sensitivity									3%
**Overall Adverse Score**	0	0.3	0.1	0	0.1	0.1	0	0.2	0
**Symptoms-Adverse**									

General worsening, no one specific symptom		1%	1%		1%	1%			
Aggression/Agitation			1%			2%			
Gastrointestinal problems		17%	2%		3%	2%			
Irritability					1%				
Loss of Appetite		1%							

Table S6. Individual Vitamins/Vitamin-like Nutraceuticals. The table lists the Overall Benefit Score, the most common benefits, the Overall Adverse Score, and the most common adverse effects.

	**Individual Vitamins/ Vitamin-like Supplements**																					
	**Treatments (n)**																					
	**Folinic Acid, high dose (21)**	**Vitamin C (182)**	**Vitamin D (159)**	**Folinic Acid, moderate dose (25)**	**P5P (25)**	**Vitamin B12 (Oral) (152)**	**Vitamin B12 (Injected) (99)**	**Coenzyme Q10 (CoQ10) (68)**	**Folic Acid (25)**	**Vitamin A (not as cod liver oil) (34)**	**Vitamin E (42)**	**Vitamin A (as cod liver oil) (31)**	**Vitamin B1 (34)**	**Vitamin B3 (26)**	**Biotin (34)**	**Vitamin B2 (29)**	**Vitamin K (29)**	**Inositol (21)**	**Vitamin B5 (20)**	**Vitamin B6, medium dose (40)**	**DMG (45)**	**TMG (28)**
**Overall Benefit Score**	2.2	1.9	1.9	1.8	1.8	1.8	1.8	1.6	1.6	1.6	1.6	1.5	1.5	1.5	1.4	1.4	1.4	1.3	1.3	1.3	1.2	1
**Symptoms-Benefit**																						
General Benefit, no one particular symptom	52%	57%	52%	56%	40%	40%	32%	63%	60%	59%	60%	56%	62%	62%	44%	59%	48%	14%	52%	43%	31%	18%

Aggression/ Agitation																						
Anxiety					20%				8%					8%				14%				
Attention	29%				16%		20%				10%	6%	3%	8%		7%	3%			13%		
Cognition	33%					25%	28%	16%								7%				8%	9%	
Constipation		12%																				
Depression			6%																			
Eczema/Skin Problems															9%							
General health																			10%			
Health		27%	18%							6%	14%	10%	6%				10%					
Hyperactivity									4%						6%							
Irritatbility																		10%				
Language/Communication	24%			20%		18%	30%														11%	29%
Lethargy								9%														
Social Interaction				16%																		11%
Stimming/Perseveration /Desire for Sameness										9%												

**Overall Adverse Score**	0.3	0.1	0	0.2	0.2	0.2	0.3	0	0.1	0.1	0	0.1	0.09	0.2	0	0.07	0	0.2	0	0.1	0.2	0.3
**Symptoms-Adverse**																						
General worsening, no one specific symptom				4%	4%								3%									
Aggression/ Agitation		1%		4%	4%	3%	4%			3%					3%			5%		3%	2%	
Anxiety	10%			4%		3%												5%				4%
Behavior Problems										3%								5%				
Decreased Cognition												3%										
Dry Mouth															9%							
Gastrointestinal Problems		2%													6%	3%						
Headache/ Migraine																				3%		

Hyperactivity	10%		1%		8%	3%	7%		8%				3%	4%		3%					7%	11%
Irritability	10%						7%			3%		3%									7%	7%
Liver/Kidney Problem		2%																				
Stimming/Perseveration/Desire for Sameness			1%																			

Table S7. Multivitamins. The table lists the Overall Benefit Score, the most common benefits, the Overall Adverse Score, and the most common adverse effects.

	**Multivitamins**		
	**Treatments (n)**		
	**High Dose Multivitamin (46)**	**High Dose Multivitamin for Children/Adults with ASD (103)**	**Generic Child/Adult Multivitamin (436)**
**Overall Benefit Score**	1.9	1.8	1.4
**Symptoms-Benefit**			
General benefit, no one particular symptom	52%	50%	55%
Attention		19%	
Cognition	13%	21%	2%
Health	26%		10%
**Overall Adverse Score**	0.2	0.2	0
**Symptoms-Adverse**			
General worsening, no one specific symptom	2%	2%	0.2%
Aggression/Agitation			0.5%
Gastrointestinal Problems	2%	2%	
Hyperactivity		3%	0.5%
Irritability	2%		

Table S8. Sleep Treatments. The table lists the Overall Benefit Score, the most common benefits, the Overall Adverse Score, and the most common adverse effects.

	Sleep Treatments		
	Treatments (n)		
	**Melatonin (367)**	**5-HTP (45)**	**Valerian Root (38)**
**Overall Benefit Score**	2.1	1.8	1.2
**Symptoms-Benefit**			
General benefit, no one particular symptom	6%	27%	
Falling asleep	74%	36%	53%
Irritability			11%
Staying asleep	35%	27%	29%
**Overall Adverse Score**	0.3	0.1	0
**Symptoms-Adverse**			
Aggression/Agitation	2%	4%	5%
Anxiety		2%	
Behavior Problems	2%		5%
Fatigue/Drowsiness			5%
Headache/Migraine		2%	
Sleep Problems	7%		

Table S9. Other Miscellaneous Nutraceuticals. The table lists the Overall Benefit Score, the most common benefits, the Overall Adverse Score, and the most common adverse effects.

	**Other Nutraceuticals**				
	**Treatments (n)**				
	**Fruit/Vegetable Powder Concentrate (21)**	**Epsom Salt (141)**	**Milk Thistle (28)**	**Carnitine (54)**	**GABA (69)**
**Overall Benefit Score**	2.2	1.6	1.5	1.3	1.3
**Symptoms-Benefit**					
General Benefit, no one particular symptom	67%	22%	46%	43%	25%
Aggression/Agitation		35%	4%		
Anxiety					26%
Attention		26%			
Cognition				9%	
Constipation	24%				
Falling Asleep					17%
Health	24%				
Irritability			7%		
Lethargy				19%	
**Overall Adverse Score**	0	0	0	0.1	0.2
**Symptoms-Adverse**					

General worsening, no one specific symptom		1%			
Aggression/Agitation			4%	6%	6%
Anxiety		1%	4%		4%
Behavioral Problems					4%
Hyperactivity			4%		
Irritability				6%	
Rash		1%			
